# Eosinophilic Orbital Myositis Superseding Ocular Myasthenia

**DOI:** 10.31138/mjr.150523.eom

**Published:** 2024-01-31

**Authors:** Georges El Hasbani, Ali Tarhini, Razane Wehbe, Diamond Ghieh, Lama Farhat, Imad Uthman

**Affiliations:** 1 Department of Medicine, Hartford Healthcare St. Vincent’s Medical Centre, Bridgeport, CT, USA,; 2 Faculty of Medicine, American University of Beirut, Beirut, Lebanon,; 3 Department of Diagnostic Radiology, American University of Beirut Medical Centre, Beirut, Lebanon,; 4 Department of Pathology and Laboratory Medicine, American University of Beirut Medical Centre, Beirut, Lebanon,; 5 Department of Internal Medicine, American University of Beirut Medical Centre, Beirut, Lebanon

**Keywords:** ocular eosinophilic myositis, associations, Graves’ disease

## Abstract

Various muscles can be involved in idiopathic eosinophilic myositis (IEM), with the ocular muscles being notably affected. Ocular eosinophilic myositis is a rare condition that typically affects the rectus muscles. A tissue biopsy stands as the gold standard for diagnosis. Different subtypes exist based on the extent of eosinophilic infiltration. Limited data is available about treatment, although glucocorticoids have shown successful outcomes. We present the case of a 60-year-old man who, a few years after being diagnosed with ocular myasthenia gravis, was diagnosed through a tissue biopsy with ocular eosinophilic myositis. Treatment with oral glucocorticoids significantly improved his symptoms.

## INTRODUCTION

Idiopathic eosinophilic myositis (IEM) is an uncommon disorder characterised by eosinophilic infiltration into the muscles.^1^ This infiltration can lead to a maladaptive response affecting T cells, B cells, and plasma cells.^2^ The true cause of IEM is still unknown, although certain genetic abnormalities have been described.^3^ Despite clinical indicators such as myalgias and laboratory findings such as elevated creatine kinase or absolute eosinophil count, diagnosing the condition typically requires a muscle biopsy.^4^

Histologically, IEM may be divided into three categories; focal eosinophilic myositis, diffuse eosinophilic myositis, and eosinophilic perimyositis.^5^ The most frequently affected muscles are the striated skeletal muscles, including those in the oesophagus and heart.^3^ Involvement of orbital muscles has been exceptionally rare. This occurrence has been described in connection with other immune-mediated diseases such as eosinophilic granulomatosis with polyangiitis (EGPA)^6^ and myasthenia gravis.^7^

This case report emphasises the development of eosinophilic orbital myositis in a 60-year-old man previously diagnosed with ocular myasthenia gravis. Treatment with oral glucocorticoids resulted in the resolution of symptoms and the normalisation of laboratory values.

## CASE DESCRIPTION

A 60-year-old man, with a significant medical history of diabetes mellitus managed with metformin and a recent HbA1c of 6.2%, presented with symptoms of blurred vision, ptosis, and diplopia affecting both eyes, leading to the diagnosis of open-angle glaucoma. Treatment was initiated with Dorzolamide-Timolol eye drops.

Despite this treatment, persistent symptoms prompted the performance of a single fibre electromyography (EMG) of the orbicularis oculi muscle, revealing findings consistent with new-onset ocular myasthenia gravis. Positive results for Anti Acetylcholine receptor antibodies at 6.2 nmol/L supported the diagnosis. The patient received a one-time dose of Rituximab and started prednisolone therapy, resulting in the resolution of symptoms.

**Figure 1.**
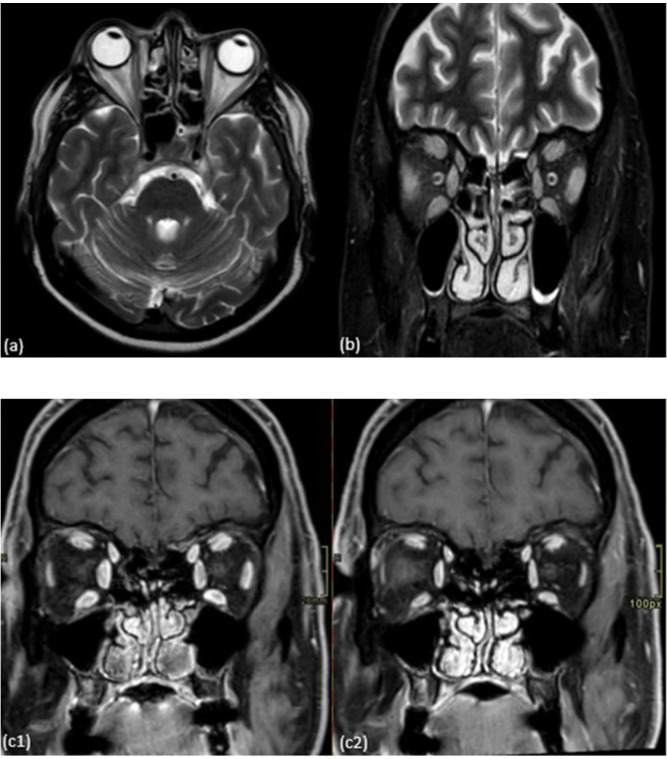
**(a)** MRI of the brain. **(b)** Axial T2W images of the brain demonstrates bilateral proptosis, right more than left. **(c)** Coronal STIR images through the orbits show increased signal intensity within the bilateral intraocular muscles suggestive of oedema. Coronal post contrast SPIR images of the orbits showing increased thickness of the intraocular muscles in October 2022 (**c1**) compared to images from April 2017 (**c2**).

**Figure 2.**
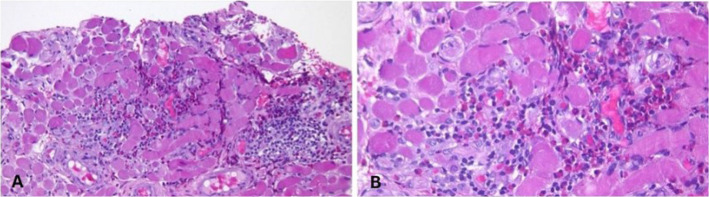
Groups of lymphocytes (white arrow) and eosinophils (black arrow) in muscle tissue. **(a)**. power 20 magnification. **(b)** Power 40 magnification.

After a few years, the patient reported bilateral ocular allergic symptoms, including swollen lids and chemosis, accompanied by significantly elevated eosinophilia (28%). Pharmacological therapy with antihistamines and glucocorticoids failed to resolve the symptoms. A magnetic resonance imaging (MRI) of the orbit showed bilateral proptosis and enlargement of intraocular muscles, notably more pronounced on the right (**Figure 1**). A tissue biopsy from the right eye revealed changes in the medial rectal, inferior rectal, and inferior oblique muscles, suggestive of orbital eosinophilic myositis (**Figure 2**). Consequently, the patient was started on a daily oral dose of prednisolone (20 mg), leading to complete resolution of symptoms and normalisation of eosinophil levels. Further investigations, including a fine needle aspirate and biopsy of the bone marrow, showed no evidence of any malignant or benign aetiology.

## DISCUSSION

Idiopathic eosinophilic myositis can be broadly categorised into three distinct types that differ histologically, clinically, and radiologically: diffuse eosinophilic myositis, eosinophilic myofasciitis, and eosinophilic fasciitis.^4^ Diffuse eosinophilic myositis is characterised by eosinophil infiltration into the muscle, excluding the fascia. It typically presents with muscle weakness, often observed in individuals over the age of 70.^5^ On the other hand, patients with eosinophilic fasciitis, also predominantly elderly, do not exhibit muscle weakness as the inflammatory changes primarily involve the fascia rather than the muscles.^8^ Conversely, individuals with eosinophilic myofasciitis, typically younger, do not display objective muscle weakness. Eosinophils infiltrate both the muscles and the fascia.^8^ In the case of our patient, muscle involvement without fascial changes indicates a diagnosis of diffuse eosinophilic myositis. The relationship between diabetes mellitus, present in our patient, and the likelihood of developing Idiopathic eosinophilic myositis remains unclear.

The involvement of extraocular muscles in eosinophilic myositis is exceedingly rare. **Table 1** lists reported muscles typically affected. Notably, previous cases have not reported the involvement of the oblique muscles or levator palpebrae superioris.

**Table 1.** Extraocular muscle involvement in eosinophilic myositis.

**Ocular muscles involved**	**References**
**Superior rectus**	^6, 7, 13^
**Inferior rectus**	^7^
**Lateral rectus**	^6, 7, 13^
**Medial rectus**	^7, 14^

Ocular eosinophilic myositis has been reported in association with certain autoimmune diseases. For instance, myasthenia gravis is one of the conditions that could coexist with eosinophilic myositis, both potentially contributing to muscle weakness.^9^ When present together, IEM typically supersedes the diagnosis of myasthenia gravis.^10,11^ Eosinophil count can aid in differentiating symptoms that may indicate a myasthenia flare. Additionally, a muscle biopsy remains the gold standard for accurate differentiation. Eosinophilic myositis has also been associated with eosinophilic granulomatosis with polyangiitis.^6,12^

## CONCLUSION

Involvement of the ocular muscles by eosinophilic myositis, referred to as ocular eosinophilic myositis, remains a rare occurrence. This condition primarily affects the rectus muscles, with less frequent involvement of the oblique muscles. Certain autoimmune diseases, such as myasthenia gravis, have been linked to ocular eosinophilic myositis. Typically, myasthenia gravis precedes the diagnosis of ocular eosinophilic myositis, although the underlying reasons for this temporal relationship are yet to be fully understood.
